# A Statistical Framework for Accurate Taxonomic Assignment of Metagenomic Sequencing Reads

**DOI:** 10.1371/journal.pone.0046450

**Published:** 2012-10-01

**Authors:** Hongmei Jiang, Lingling An, Simon M. Lin, Gang Feng, Yuqing Qiu

**Affiliations:** 1 Department of Statistics, Northwestern University, Evanston, Illinois, United States of America; 2 Department of Agricultural and Biosystems Engineering, University of Arizona, Tucson, Arizona, United States of America; 3 Interdisciplinary Program in Statistics, University of Arizona, Tucson, Arizona, United States of America; 4 Biomedical Informatics Research Center, Marshfield Clinic Research Foundation, Marshfield, Wisconsin, United States of America; 5 Institute for Clinical and Translational Research, University of Wisconsin at Madison, Madison, Wisconsin, United States of America; 6 Biomedical Informatics Center, Northwestern University, Chicago, Illinois, United States of America; Indiana University, United States of America

## Abstract

The advent of next-generation sequencing technologies has greatly promoted the field of metagenomics which studies genetic material recovered directly from an environment. Characterization of genomic composition of a metagenomic sample is essential for understanding the structure of the microbial community. Multiple genomes contained in a metagenomic sample can be identified and quantitated through homology searches of sequence reads with known sequences catalogued in reference databases. Traditionally, reads with multiple genomic hits are assigned to non-specific or high ranks of the taxonomy tree, thereby impacting on accurate estimates of relative abundance of multiple genomes present in a sample. Instead of assigning reads one by one to the taxonomy tree as many existing methods do, we propose a statistical framework to model the identified candidate genomes to which sequence reads have hits. After obtaining the estimated proportion of reads generated by each genome, sequence reads are assigned to the candidate genomes and the taxonomy tree based on the estimated probability by taking into account both sequence alignment scores and estimated genome abundance. The proposed method is comprehensively tested on both simulated datasets and two real datasets. It assigns reads to the low taxonomic ranks very accurately. Our statistical approach of taxonomic assignment of metagenomic reads, TAMER, is implemented in R and available at http://faculty.wcas.northwestern.edu/hji403/MetaR.htm.

## Introduction

Traditional and classical methods of genomics and microbiology allow researchers to study an individual microbial species obtained from the environment by isolating the organism into pure colonies using microbial culture techniques. However, this approach cannot capture the structure of the broader microbial community within the environmental sample, the relative representation of multiple genomes, and their interaction with each other and with the environment. Additionally, a large number of microbial species are very difficult, or impossible, to culture *in vitro* in the laboratory setting. The development of next-generation sequencing has advanced the field of metagenomics by enabling scientists to simultaneously study multiple genomes recovered directly from an environmental sample, thereby bypassing the need for microbial isolation through culturing (see [1] for a review).

In a metagenomic experiment, a sample is usually taken from a natural (e.g., soil and seawater) or a host-associated (e.g., human gut) environment containing micro-organisms organized into communities or microbiomes. DNA is extracted from the environmental sample containing a mixture of multiple genomes and then sequenced without prior separation. The resulting dataset comprises millions of mixed sequence reads from the multiple genomes contained in the sample. Traditionally, DNA has been sequenced using Sanger sequencing technology [2] and the reads generated are routinely 800–1000 base pairs long. However this technology is extremely cumbersome and costly. Recently next-generation sequencers, e.g., Illumina/Solexa, Applied Biosystems' SOLiD, and Roche's 454 Life Sciences sequencing systems, have emerged as the future of genomics with incredible ability to rapidly generate large amounts of sequence data [3], [4]. These new technologies greatly facilitate high-throughput while lowering the cost of metagenomic studies. However, the reads generated are of much shorter length making reads assembly and alignment more challenging. For example, Illumina/Solexa and SOLiD generate reads ranging between 35–100 base pairs while Roche 454 reads are approximately 100–400 base pairs in length.

One goal of metagenomic studies is to identify what genomes are contained in the environmental sample and to estimate their relative abundance. Identification of genomes is complicated by the mixed nature of multiple genomes in the sample. A widely used approach is assigning the sequence reads to NCBI’s taxonomy tree based on sequence read homology alignment with known sequences catalogued in reference databases. The sequence reads are first aligned to the reference sequence databases using a sequence comparison program such as BLAST [5]. Reads which have hits in the database are then assigned to the taxonomy tree based on the best match or multiple high-scoring hits. The challenge of this approach is that hits may be found in multiple genomes for a single read at a given threshold of bit-score or Expect value, due to sequence homology and overlaps associated with similarity among species. Strategy of weighting similarities for multiple BLAST hits has been used to estimate the relative genomic abundance and average size [6]. Another representative and stand-alone analysis tool, MEGAN [7], assigns a read with hits in multiple genomes to their lowest common ancestor (LCA) in the NCBI taxonomy tree. Thus assignments of reads to different ranks of taxonomy tree depend on what threshold for bit-score or Expect value is used. Furthermore, MEGAN assigns reads one at a time. As a consequence, the results have less false positives but lack specificity. Various methods have been proposed to improve the taxonomic assignment of reads by assigning more reads to the lower ranks of taxonomy tree [8]–[12]. In particular, CARMA3 [10] which is BLAST-based but not LCA-based, uses reciprocal search technique as in SOrt-ITEMS [13] to reduce the number of hits and hence further improves the accuracy of the taxonomic classification.

In this paper, we propose a statistical approach, TAMER, for taxonomic assignment of metagenomic sequence reads. In this approach we first identify a list of candidate genomes using homology searches. A mixture model is then employed to estimate the proportion of reads generated by each candidate genome. Finally, instead of assigning reads one at a time to the taxonomy tree as done by LCA-based methods, reads are assigned to the genomes in a global manner by taking into account both sequence alignment scores and estimated proportion of reads generated by each genome. The proposed method is comprehensively tested on simulated metagenomic data with diverse complexity of microbial community structure and with various read length and also applied to several real world metagenomic datasets. Compared with other available algorithms and tools designated for metagenomic analysis, the proposed approach demonstrates greater accuracy in identification and quantification of multiple genomes in a given sample.

## Materials and Methods

### Input Data

The proposed homology-based method, TAMER, identifies multiple genomes based on the hits obtained by aligning sequence reads against known reference sequence databases. In this paper we use the NCBI-NT instead of NCBI-NR database as reference sequence source in our data analysis. NT database contains almost all known nucleotide sequences of all known species from NCBI GeneBank, EMBL and DDBJ, while NR database does not have reference sequences for reads generated from intergenic regions. Although BLAST is the traditional sequence comparison and alignment tool for the NT database, computation time is the bottleneck [7], [9]. High performance computing infrastructure and fast alignment tools such as BLAT [14] have been recommended when dealing with large megagenomic datasets [15]. The alignment tools developed especially for next generation sequencing technologies retrieve matches with high similarities. In this research we use MegaBLAST (version 2.2.25+) which yields matches with relatively high similarities but is much faster than BLASTn [16]. Notably, our proposed method has great versatility and can also be applied when other alignment tools and other reference databases are used.

When aligning reads to the reference database, there may be multiple hits within one genome for a sequence read. In this situation, the hit with the largest number of identical matches is chosen to represent the corresponding genome. For each read and the corresponding hits in one or multiple genomes, we record the genome name or taxon identification number of the hit, number of matched base pairs, and the alignment length. These consist of the input data for the proposed method which evaluates the likelihood of alignment of a read with a given genome among the list of candidate genomes.

### Mixture Model

Suppose we have a total of n sequence reads 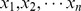 which have been aligned to *K* candidate genomes. For each of the *K* genomes, there is at least one sequence read having hit. Let 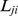 denote the alignment length and 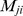 be the number of matched base pairs when aligning read 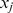 against genome 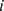. If a read 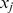 does not have any hit in genome 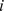, then 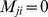. Due to differences in genome compositions, a short read is usually aligned to one or a few genomes. Thus, the scoring matrix *M* below, where rows represent reads and columns represent genomes, is very sparse, i.e., most entries in the matrix are zero.
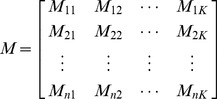
(1)


To identify which of the *K* candidate genomes in the scoring matrix are truly contained in the metagenomic sample, we propose a statistical framework to model the matches between the reads and reference sequences. Let 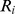 denote the proportion of reads generated from genome 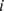
(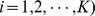, where 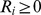 and the sum of 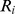 is 1. If the reads are randomly generated by the *K* genomes, then the probability that a read 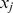 is generated by genome 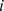 is 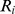. Even if a read 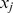 is generated from genome 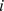, it is possible that the match is not 100% identical due to sequencing errors, alignment errors, and/or single nucleotide polymorphism (SNP). Let *p* denote the probability of observing a mismatched base pair, then 1- *p* is the probability of observing a matched base pair. The probability that a read 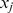 is generated by genome 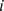 with 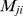 matched base pairs and 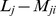 mismatched base pairs is 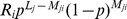, where 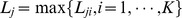 is the maximum alignment length. Then the probability of observing a read 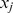 in the dataset is
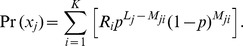



Assuming that the reads are independent of each other, the likelihood function of the data is:
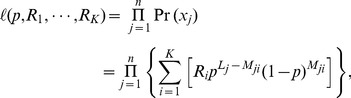
(2)where the values of 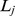 and 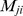 are observable, and the parameters *p* and 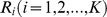 are to be estimated.

### EM Algorithm

For this mixture model, the expectation maximization (EM) algorithm [17] is used to calculate the maximum likelihood estimation for the parameters *p* and 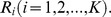 Let 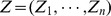 be the latent variables that determine the genome from which each read originate. The aim is to estimate the unknown parameters 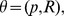 where 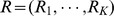. The likelihood function can be written as:
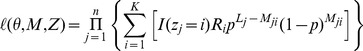
where 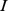 is an indicator function. As the density function is an exponential family function, the likelihood function can be expressed as:



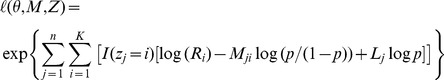

- **Initialization step.** Initialize the values of *p* and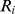 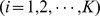, call them 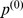 and 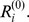 For instance, let the reads be equally distributed among the K genomes, i.e., 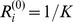, and let 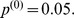

- **E-step.** Assuming the current estimate of the parameter is 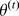, then the conditional distribution of 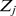 is:

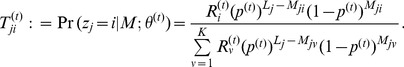
(3)Then the E-step result is:
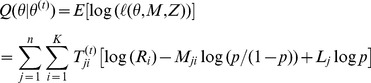

- **M-step.** As the parameters can be maximized independently, we get:

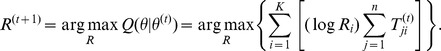



This gives 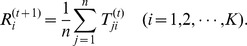


The probability of observing a mismatched base pair is estimated as:
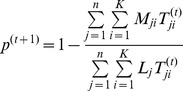

- **Iteration step.** Repeat the E-step and the M-step until all the parameters converge, i.e., 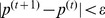 and 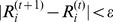 for 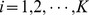 and for some pre-specified small number of ε.

The estimates of 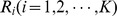 reflect the proportion of reads generated from each of the *K* candidate genomes. If 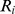 =  0, then the corresponding genome 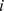 is not contained in the sample. If we observe an inequality 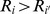 for two genomes 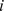 and 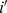, then we conclude that the sample contains more reads generated from genome
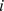
than genome 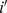. However the values of 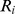 do not give information on which reads are generated by which genomes. Next we show how to assign reads to the *K* candidate genomes and the taxonomy tree.

### Taxonomic Assignment of Reads

To assign each read to the taxonomic tree, we first estimate how likely it is generated by a specific genome. The probability that read 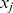 is generated by genome 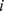 is estimated by.
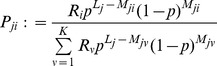
for 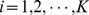 and 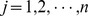. Then read 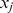 is assigned to the genome for which the maximum probability is reached, i.e., read 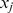 is assigned to genome 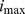 where 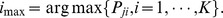 An assignment matrix 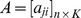 can be constructed based on the read assignment, where 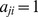 if read 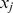 is assigned to genome 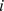, and 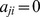 otherwise. Then the total number of reads assigned to genome 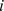 is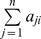.

The proposed method, TAMER, applies to the candidate genomes to which the sequence reads have hits. Note the majority of the candidate genomes identified after performing BLAST are at the low ranks of the taxonomy tree, i.e., most of the genomes are species or substrings of species. Once a read is assigned to a specific genome, we also consider that it is assigned to taxa with higher taxonomic ranks. For example, suppose a read is assigned to *Escherichia coli str. K-12 substr. MG1655.* When we summarize reads assigned at different taxonomic ranks, this read is treated as that it is assigned to *Escherichia. coli* at rank Species, to *Escherichia* at rank Genus, to *Enterobacteriaceae* at rank of Family, and so on.

### Estimates of Relative Genome Abundance

The number of sequence reads generated by a genome is proportional not only to the number of copies of that genome in the metagenomics sample but also to the length of the genome [6]. Similar to [18], the relative genome abundance can be computed for known genomes which are present in the sample. Let 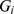
denote the actual length of the genome
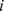
in base pairs. Suppose there are 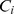
copies of genome
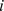
in the sample. Assuming uniform distribution of reads across the multiple genomes, we have.
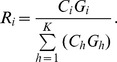



Then the relative abundance of genome 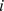 (i.e., relative copy number) in the sample can be calculated by.
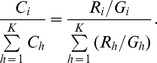



### Algorithm Implementation

All algorithms developed in this research are implemented in R, a free software environment for statistical computing and graphics [19]. The R source codes are available at http://faculty.wcas.northwestern.edu/hji403/MetaR.htm. For practical implementation, the scoring matrix *M* in equation (1) could require a huge storage space when the total number of reads is large. Recognizing that *M* is a sparse matrix, substantial memory requirement reductions can be achieved by storing only the non-zero matching scores. For the zero entries of 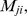
their influence on estimating the parameters is nominal because we have 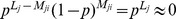
when 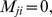 for a small value of 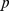
(e.g., 0.02∧35 = 3.4e-60). With the use of sparse matrix technique, detecting multiple genomes via the mixture model becomes very efficient. For example, the computational time for a dataset of 150,000 reads with average read length of 100 bp is about 2 ∼ 3 minutes on a laptop with 8 GB RAM and 2 core 3.06 GHz CPU.

### Simulation Studies

Due to the complexity of metagenomic data, simulation studies with verifiable results are crucial to benchmark TAMER and conduct comparisons with other existing methods. For the analysis by MEGAN the default parameters are used.

#### Simulation study 1

MetaSim [20], a sequencing simulator for genomics and metagenomics, is used to generate sequence reads for simulation studies. Four benchmark simulation datasets with low (2 genomes, simLC), medium (9 genomes, simMC), high (11 genomes, simHC), and super high (100 genomes, simSC) complexity are used. The first three setups were designed by [20] in conjunction with [21]. We use the simulation study with 100 genomes to reflect the high complex structure of some microbial communities which may contain hundreds even thousands of species. Here, we present the simulation studies using reads with an average read length of 100 bp for all four simulation studies, thereby mimicking next-generation sequencing short reads. For the medium and high complexity, we also perform simulation study using average read length of 400 bp. In this simulation study, we compare the performance of TAMER with MEGAN.

#### Simulation study 2

To compare TAMER with CARMA3 [10], we use the same evaluation dataset as in [10]. This CARMA3 evaluation dataset consists of 25,000 metagenomic reads which are randomly simulated from 25 bacterial genomes with an average read length of 265 bp. The online version of CARMA3, WebCARMA (http://webcarma.cebitec.uni-bielefeld.de/), with default parameters is used for taxonomic classification. We also perform the taxonomic analysis using TAMER and MEGAN, and compare their performance with CARMA3. When BLASTx and NR database are used, CARMA3 gives better taxonomic assignment than MEGAN [10]. Therefore we only present the results by MEGAN using MegaBLAST and NT database in this study.

### Real Datasets

TAMER is also applied to two sets of actual metagenomic data. Archived metagenomic datasets are accessible from several sources including the NCBI short read archive [22], CAMERA [23], and the MG-RAST server [24]. In this paper we analyze data from eight oral samples and two seawater samples.

The eight oral samples downloaded from the MG-RAST server were examined in a human metagenome oral cavity study [25]. They represent different degrees of oral health with two samples for each of the four status, healthy controls (never with caries), treated for past caries, active caries, and cavities. There are totally about 2 million reads. The smallest sample has about 70,000 reads and the largest sample has about 465,000 reads. The average read length is 425±117 bp.

The two seawater datasets were retrieved from MEGAN database (http://www.megan-db.org/megan-db/) and were studied in [20]. Each dataset consists of 10,000 reads and they are part of the Sargasso Sea Samples studied in [26]. The reads are about 800 bp long in both seawater datasets.

## Results

### Results for Simulation Study 1

Using the same abundance setup as in [20], 150,000 reads are generated for each of the three complexity datasets, simLC, simMC, and simHC, with average length of 100 bp. For the simSC dataset, 100 genomes with the same abundance are randomly selected and 150,000 reads are generated. The characteristics of the datasets are listed in Table S1. For this simulation study, we compare TAMER with MEGAN. The proportions of reads correctly (TP) and incorrectly (FP) assigned at different taxonomy ranks are reported in Table 1. Here TP = number of correctly assigned reads / total number of reads×100, and FP = number of incorrectly assigned reads/ total number of reads×100. For instance, for the simLC data, 146,880 reads are assigned to the corresponding species correctly, and 30 reads are assigned incorrectly, then TP = 146,880/150,000×100 = 97.92 and FP = 30/150,000×100 = 0.02. Note that the sum of TP and FP is not 100 as some reads do not have hits in the reference database.

**Table 1:** Results for simulation study 1 with average read length o1 100 bp.

	simLC				simMC				simHC				simSC			
	TAMER		MEGAN		TAMER		MEGAN		TAMER		MEGAN		TAMER		MEGAN	
	TP	FP	TP	FP	TP	FP	TP	FP	TP	FP	TP	FP	TP	FP	TP	FP
Species	97.92	0.02	84.74	0.01	96.14	0.86	67.57	0.83	96.97	0.15	91.97	0.00	95.70	1.20	86.05	0.11
Genus	97.92	0.00	85.02	0.01	96.17	0.76	72.95	0.86	97.00	0.03	93.59	0.00	95.87	0.70	91.67	0.00
Family	97.93	0.00	96.68	0.01	96.91	0.02	95.13	0.01	97.01	0.02	96.59	0.00	94.71	0.02	93.63	0.00
Order	97.93	0.00	96.69	0.01	96.91	0.02	95.20	0.01	97.01	0.02	96.74	0.00	95.36	0.01	94.37	0.00
Class	97.93	0.00	96.79	0.01	96.93	0.00	95.44	0.01	82.85	0.00	82.64	0.00	92.10	0.00	91.21	0.00
Phylum	97.93	0.00	96.87	0.01	96.93	0.00	95.51	0.01	97.03	0.00	96.92	0.00	96.16	0.00	95.35	0.00
Kingdom	97.94	0.01	96.98	0.01	96.99	0.00	95.66	0.01	97.11	0.00	96.97	0.00	96.90	0.00	96.19	0.00

The percentage of correctly (TP) and incorrectly (FP) assigned reads out of total 150,000 reads with average read length of 100 bp at different taxonomic ranks using TAMER and MEGAN for the simLC, simMC, simHC and simSC datasets in simulation study 1.

**Figure 1:**
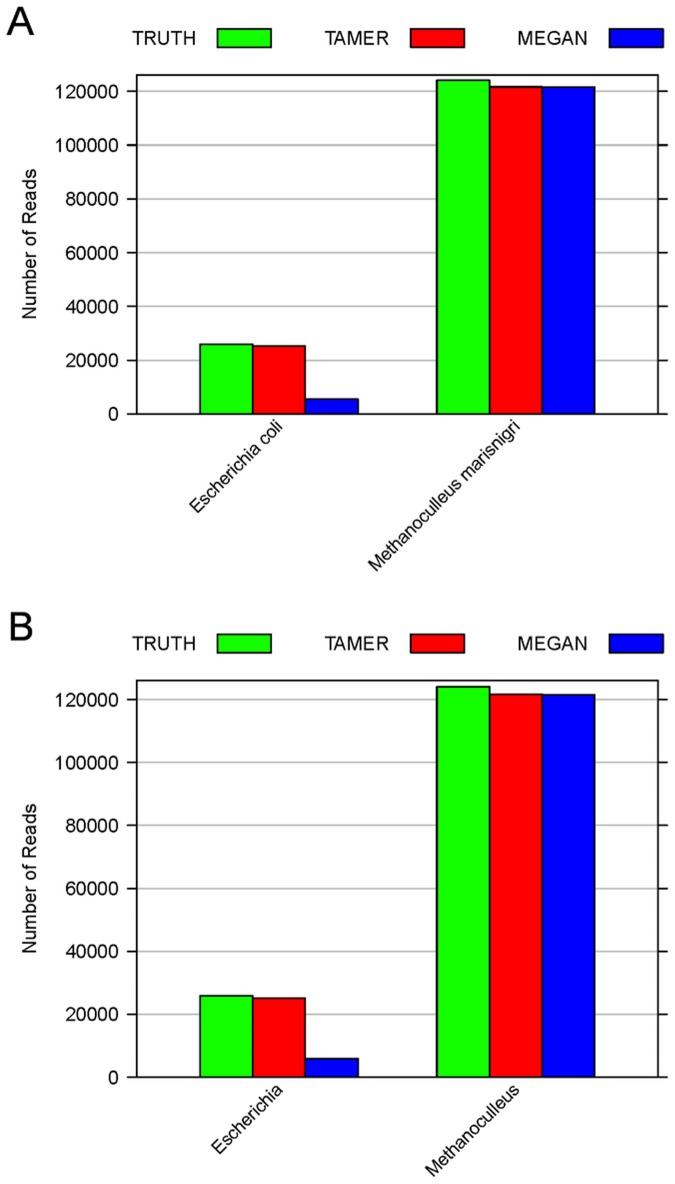
Reads assignment at rank Species and Genus for simLC dataset. Numbers of reads assigned to rank (A) Species and (B) Genus using TAMER and MEGAN are compared with the true values (TRUTH) for the simLC dataset with 150,000 reads and average read length of 100 bp in simulation study 1.

The simLC dataset consists of 25,926 reads generated from *E. coli str. K-12 substr. MG1655* and 124,074 reads generated from *Methanoculleus marisnigri JR1.* Totally there are about 160 million base pairs and the simulated error rate is 0.027. The estimated probability of observing a mismatched base pair is 0.025 by TAMER. Using MegaBLAST, hits are found for 97.94% of the 150,000 reads in 4,407 unique taxa. At rank Species, TAMER accurately assigns 25,221 reads to species *Escherichia coli* which is close to the true value of 25,926 reads, while MEGAN only assigns 5,583 reads to this taxon (Figure 1 (a)). At rank Genus, MEGAN assigns 5,974 reads to *Escherichia* which is only about 23% of the true value and the number of reads assigned by TAMER to that genus (Figure 1 (b)). Considering the low proportion of incorrect assignment (Table 1), TAMER accurately identifies and quantifies the different genomes at low taxonomic ranks.

**Figure 2:**
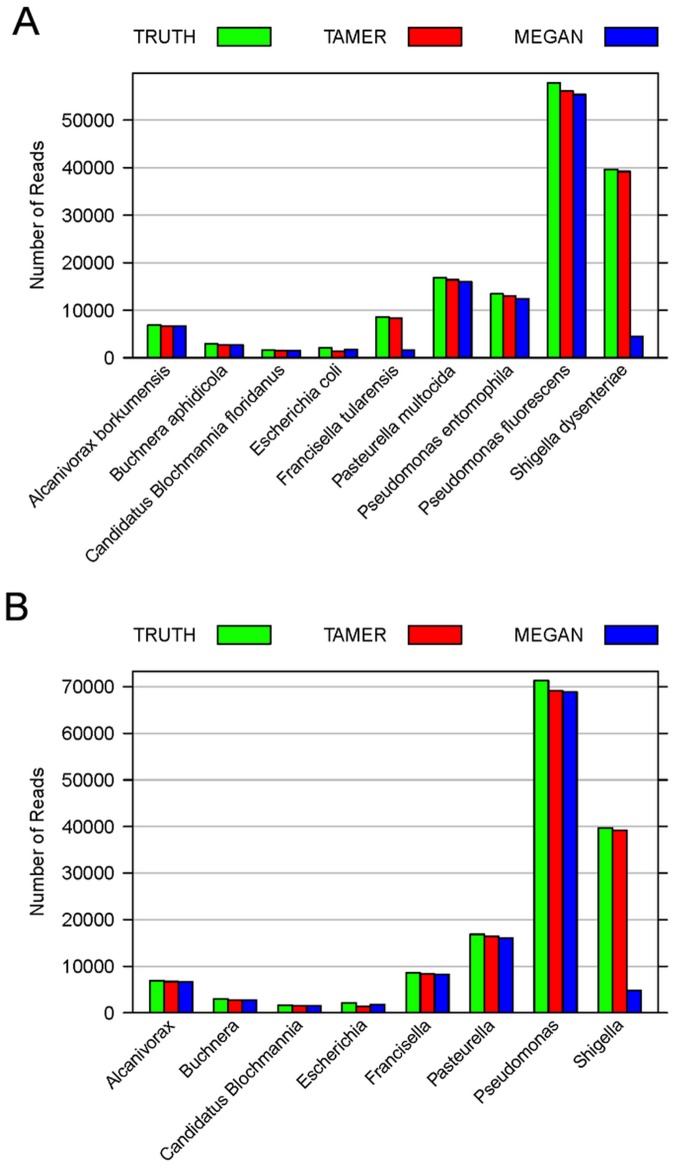
Reads assignment at rank Species and Genus for simMC dataset. Numbers of reads assigned to rank (A) Species and (B) Genus using TAMER and MEGAN are compared with the true values (TRUTH) for the simMC dataset with 150,000 reads and average read length of 100 bp in simulation study 1.

The simMC dataset consists of nine microbial organisms from phylum *Proteobacteria* with diverse relative abundance. Hits are found for 97.00% of the 150,000 reads in 9,925 unique taxa. TAMER is able to dramatically reduce the huge number of taxa, and accurately identifies the nine organisms and assigns the reads to the corresponding originated organisms. TAMER assigns 96.14% of the reads correctly at rank Species, while MEGAN only assigns 67.57% of reads (Table 1). At rank Genus, the proportion of assigned reads by MEGAN is increased to 72.95%, however it is 23% less than that by TAMER. The percentage of incorrectly assigned reads is about 0.8% for both TAMER and MEGAN at both ranks of Species and Genus. It is evident that the number of reads assigned to different taxa by TAMER is very close to the true value, while MEGAN assigns 6,643 less reads to *Francisella tularensis* and 39,184 less reads to *Shigella dysenteriae* than TAMER does (Figure 2 (a)). At rank Genus, TAMER assigns 39,191 reads to *Shigella* which is close to the true value and is about eight times as many as MEGAN does (Figure 2 (b)).

**Table 2:** Results for CARMA3 evaluation dataset.

	TAMER		MEGAN		CARMA3	
	TP	FP	TP	FP	TP	FP
Species	99.24	0.73	81.45	0.02	4.57	0.12
Genus	99.26	0.68	91.52	0.03	64.10	0.43
Family	89.39	0.00	88.55	0.00	73.20	0.10
Order	97.22	0.00	96.40	0.00	83.48	0.12
Class	92.11	0.00	91.42	0.00	82.34	0.10
Phylum	99.94	0.00	99.31	0.00	90.50	0.07
Kingdom	99.97	0.00	99.42	0.00	90.90	0.12

The percentage of correctly (TP) and incorrectly (FP) assigned reads out of total 25,000 reads at different taxonomic ranks using TAMER, MEGAN and CARMA3 for the CARMA3 evaluation dataset in simulation study 2.

The simHC dataset consists of 11 microbial organisms. Using MegaBLAST, hits are found for 97.11% of 150,000 reads in 2,511 unique taxa. TAMER identifies all 11 genomes and assigns the reads accurately to the original organisms. For these 11 distantly related organisms, MEGAN also does a satisfactory work by assigning about 92% of reads at rank Species which is 5% less than TAMER does (Table 1). Population distributions of reads at rank Species (Figure S1) and Genus (Figure S2) show that the assignments of reads by both methods are similarly accurate.

**Figure 3:**
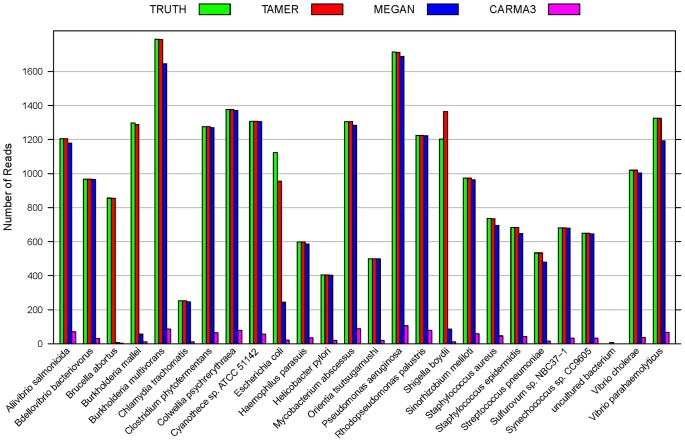
Reads assignment at rank Genus for CARMA3 dataset. Numbers of reads assigned to rank Genus using TAMER, MEGAN, and CARMA3 are compared with the true values (TRUTH) for the CARMA3 evaluation dataset in simulation study 2.

The simSC dataset is generated from 100 microbial organisms. About 96.90% of 150,000 reads have matches in 14,205 unique taxa. TAMER identifies 149 genomes with 103 of them having at least 5 assigned reads. Summarizing the results at different taxonomic ranks, TAMER assigns about 8% more reads than MEGAN at rank Species, and TAMER and MEGAN are comparable at higher taxonomic ranks (Table 1).

**Figure 4:**
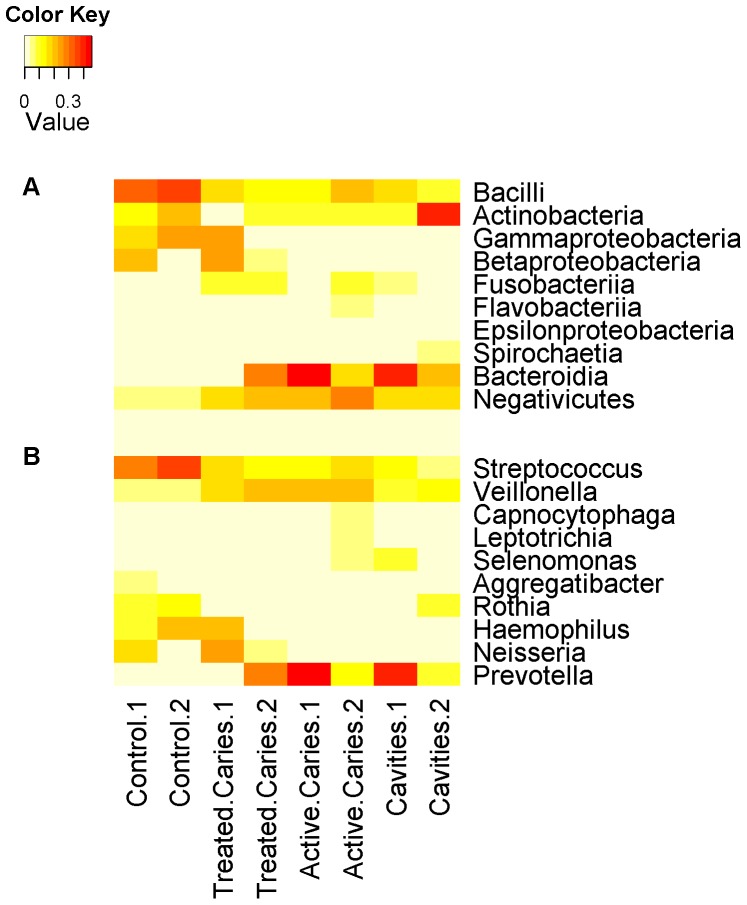
Heatmaps for oral samples. Heatmaps for the abundant (A) classes and (B) genera represent the estimated proportion of reads assigned to each of the eight samples based on TAMER.

For simMC and simHC, we also perform a simulation study using 10,000 reads with average read length of 400 bp. With longer read length, the proportion of correctly assigned reads at low taxonomic ranks is improved for both methods. This further confirms the very well-known fact that longer reads are more sensitive in estimating the relative abundance of the multiple species. For simMC data, TAMER and MEGAN assign about 99.9% and 71.4% of reads correctly at rank Species, respectively, while the proportion of incorrectly assigned reads only increases about 0.1% for TAMER (Table S2). At rank Genus, TAMER assigns about 23% more reads correctly than MEGAN (99.91% for TAMER and 76.53% for MEGAN) while the false positive rate only increases about 0.08%. For simHC simulation study, the results of TAMER and MEGAN are highly comparable.

**Table 3:** Results for the two seawater samples.

	Sample 1			Sample 2		
	TAMER	MEGAN	CARMA3	TAMER	MEGAN	CARMA3
Species	93.92	45.82	3.92	86.92	35.36	0.54
Genus	91.77	70.98	60.20	81.84	34.02	15.63
Family	75.86	59.82	54.16	57.77	18.30	5.00
Order	91.62	77.12	57.88	80.69	41.74	8.44
Class	85.57	74.40	73.71	74.10	42.81	37.66
Phylum	92.84	81.36	83.92	83.53	50.12	53.65
Kingdom	96.50	87.32	90.27	93.86	68.85	73.87

The percentage of reads out of total 10,000 reads assigned at different taxonomic ranks using TAMER, MEGAN and CARMA3, for each of the two seawater samples.

### Results for Simulation Study 2

For the CARMA3 evaluation dataset, the results based on TAMER and MEGAN are listed in Table 2, where we also list the results of CARMA3 which are reported in the original paper [10]. At rank Species, the percentage of correctly assigned reads is 99.24% for TAMER, 81.45% for MEGAN, and 4.57% for CARMA3 (Table 2). At rank Genus, the proportion of correctly assigned reads by TAMER (99.26%) is 7% and 35% more than MEGAN (91.52%) and CARMA3 (64.10%), respectively.

Consistent with the conclusions from simulation study 1, the numbers of reads assigned by TAMER are very close to the true values, the true positive rate is high, and the false positive rate is very low. TAMER gives more accurate assignments than MEGAN and CARMA3 at rank Genus (Figure 3). For example, it assigns about 14 times as many as reads to *Shigella* than MEGAN and CARMA3.

### Results for Real Data Analysis

#### Oral data

Identifying and quantifying bacterial species in the normal and diseased samples will help understand the development of dental caries. About 46% of the 2 million reads have hits and could be assigned to taxonomic ranks by TAMER. The number of identified species varies from about 700 to 1,400 across the eight samples. Totally 2,500 unique species are detected from this study, about 1,300 of them have at least 5 assigned reads, and about 400 species are shared by all samples.

Estimated proportions of reads for the dominant classes based on TAMER are shown in Figure 4 (a). Generally, normal sample contains more *Bacilli* and *Gammaproteobacteria* but less *Bacteroidia* than the diseased sample, which agrees with taxonomic assignment using MEGAN approach [25] (Figure S3). We also observe a large variation among the individual samples although the eight samples were selected with homogeneous clinical features. For instance, *Actinobacteria* is abundant in the two control samples, and depleted in the remaining samples except for one sample from within cavities where it shows high proportion. *Betaproteobacteria* is high in one of the two controls and one of the samples with treated cavities, but low in the remaining six samples. Examining the population distribution at the genus level (Figure 4 (b)), *Streptococcus* is enriched in the normal samples, *Prevotella* and *Veillonella* are associated with the disease, and *Fusobacterium* is not abundant in the disease samples. Our findings about these genera are also reported in a recent study [27] which hence further verified our results.

#### Seawater data

Using BLAST, about 97% of reads in sample 1 and 94% of reads in sample 2 have hits in the nt database and could be assigned to taxonomic ranks by TAMER. There are about 900 and 1,400 species detected in sample 1 and 2, respectively. TAMER assigns more reads than MEGAN and CARMA3 at different taxonomic ranks (Table 3). At rank Species, TAMER assigns about 50% more reads than MEGAN and about 90% more reads than CARMA3 for sample 1. *Candidatus Pelagibacter ubique* is dominant in both samples (Figures S4). In fact this organism is highly dominant in both salt and fresh water worldwide [28]. At rank Genus, the differences among the number of assigned reads using different methods become smaller. However TAMER still assigns about 18% more reads than MEGAN and about 37% more reads than CARMA3 for sample 1. The two seawater samples are characterized as differing from each other based on relative frequency with sample 1 containing more *Shewanella* and *Burkholderia* than sample 2 (Figures S5), which is consistent with previous conclusions [7], [26].

## Discussion

The term metagenomics, first appeared in publication about 10 years ago [29]. To date, many metagenomic projects have undertaken characterization of microbiomes in samples from different environments including human gut [30], seawater [26], and soil [31], due to the next generation sequencing technologies. Therefore metagenomics has a broad impact across many diverse areas including human health, ecology, environmental remediation, and agriculture. Tens of millions of sequence reads can be obtained from sequencing one sample. An enormous challenge is attaining efficient and accurate data capture and storage coupled with computational and statistical methods to mine information from these massive datasets.

In this paper, we propose a rigorous statistical model to accurately identify and quantify genomes contained in a metagenomic sample by taking into account both sequence alignment scores and relative proportion of reads generated by the genomes. Identification of multiple genomes is an important goal in metagenomic studies. When a read is assigned to the high rank of the taxonomy tree, it is difficult to differentiate what genus or species actually are, or are not contained in the sample, as a high rank of taxonomy tree usually contains many genera and species. The proposed method, TAMER, can be applied to unassembled reads directly. The uniqueness of TAMER is that it assigns reads among the candidate genomes to which the sequence reads have hits. It does not assign the reads one by one to the taxonomy tree. On the contrary TAMER fully utilizes the information available from all reads by employing the mixture model. Roughly speaking, the taxonomic assignment of a read not only depends on its matching score against a genome but also borrows strength or information from other reads in the dataset. If a read achieved a high score in only one genome, then this read would be considered genome-specific and will be assigned to the corresponding genome. After assigning reads to multiple genomes, we sum up the assigned reads at different taxonomic ranks. Different from other mixture models [18], [32], the sequencing error is considered and estimated in our proposed method. The comprehensive simulation studies demonstrate that TAMER is comparable with MEGAN at high taxonomic ranks, but TAMER assigns reads more accurately than MEGAN at Genus level or even Species level.

One limitation of the proposed method is that it is based on homology searches of the sequence reads in the reference databases. For reads generated from new genomes, they would not be included in the model if matches are not found. De novo assembly methods and deep sequencing are needed to discover new genomes.

As of future work, we can further assess the accuracy and uncertainty of the proportion of assigned reads along the taxonomy tree. The bootstrap method [33] by resampling the original sequence reads (i.e., sampling rows of the scoring matrix) with replacement can be used for the statistical inference. Subsequently, the parameters are estimated using the described EM algorithm for the bootstrap sample. By replicating this procedure, i.e., resampling and estimating a large number of times, (e.g., B = 1000 bootstraps), we are able to obtain the confidence interval for each parameter of interest. Since we construct the confidence intervals for the 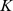 parameters 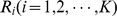 simultaneously, a multiple correction method [34] such as Bonferroni correction can be used to guarantee a pre-specified (1-α)*100% family confidence level.

## Supporting Information

Figure S1 **Barplot of the number of assigned reads by TAMER and MEGAN at rank Species for simHC data.** Numbers of reads assigned to rank Species using TAMER and MEGAN are compared with the true values (TRUTH) for the simHC data set of 150,000 reads with average read length of 100 bp.
(TIFF)

Figure S2 **Barplot of the number of assigned reads by TAMER and MEGAN at rank Genus for simHC data.** Numbers of reads assigned to rank Genus using TAMER and MEGAN are compared with the true values (TRUTH) for the simHC data set of 150,000 reads with average read length of 100 bp.
(TIFF)

Figure S3 **Scatter plot of estimated proportions byTAMER and MEGAN at different taxonomic ranks for the oral data.** Scatter plots of estimated abundance (proportion of reads) at different taxonomic ranks by MEGAN and TAMER for all eight samples.
(TIF)

Figure S4 **Population distribution of sea water samples at rank Species.** Proportions of reads assigned to the taxa at rank Species using TAMER, MEGAN and CARMA3 are compared for the sea water datasets.
(TIFF)

Figure S5 **Population distribution of sea water samples at rank Genus.** Proportions of reads assigned to the taxa at rank Genus using TAMER, MEGAN and CARMA3 are compared for the sea water datasets.
(TIFF)

Table S1 **Characteristics of data sets for simulation study 1.** Number of reads generated from each organism is listed for the simLC, simMC, simHC, and simSC datasets.
(XLS)

Table S2 **Results for simulation study 1 with average read length of 400 bp.** The percentage of correctly (TP) and incorrectly (FP) assigned reads out of total 10,000 reads with average read length of 400 bp at different taxonomic ranks using TAMER and MEGAN for simMC and simHC datasets.
(DOC)
