# Correction: A Statistical Framework for Accurate Taxonomic Assignment of Metagenomic Sequencing Reads

**DOI:** 10.1371/annotation/c08da7e7-60fe-4f7d-a457-299bf9f24148

**Published:** 2013-05-17

**Authors:** Hongmei Jiang, Lingling An, Simon M. Lin, Gang Feng, Yuqing Qiu

There was an error in the URL present at the end of the abstract. The correct link is: http://faculty.wcas.northwestern.edu/~hji403/MetaR.htm

