# Supplementary figures and images for: A Statistical Framework for Accurate Taxonomic Assignment of Metagenomic Sequencing Reads
Source: PLoS One. 2012 Oct 1;7(10):e46450. doi: 10.1371/journal.pone.0046450 (PMC3462201; doi:10.1371/journal.pone.0046450)

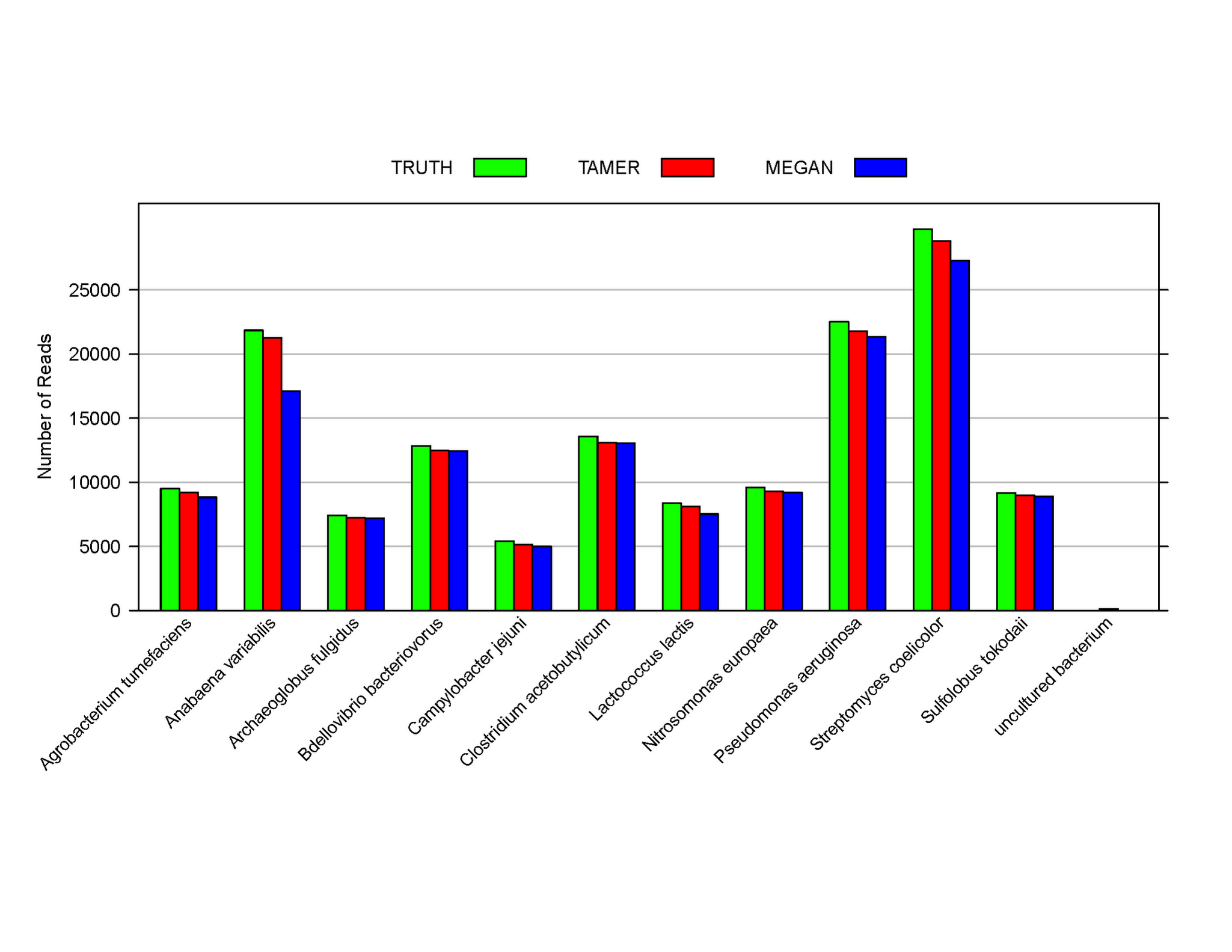

Supplement: Figure S1 — Barplot of the number of assigned reads by TAMER and MEGAN at rank Species for simHC data. Numbers of reads assigned to rank Species using TAMER and MEGAN are compared with the true values (TRUTH) for the simHC data set of 150,000 reads with average read length of 100 bp. (TIFF) [file pone.0046450.s001.tiff]

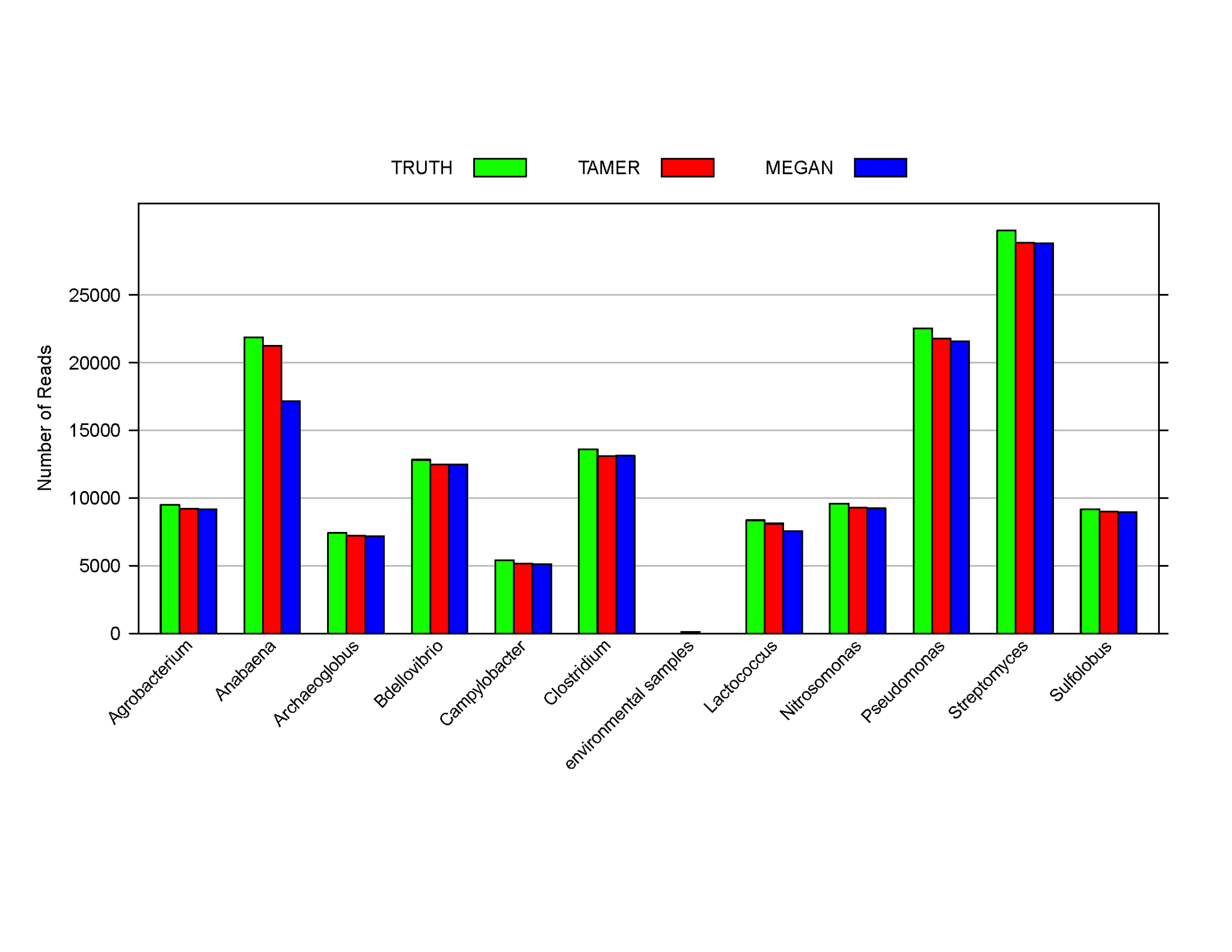

Supplement: Figure S2 — Barplot of the number of assigned reads by TAMER and MEGAN at rank Genus for simHC data. Numbers of reads assigned to rank Genus using TAMER and MEGAN are compared with the true values (TRUTH) for the simHC data set of 150,000 reads with average read length of 100 bp. (TIFF) [file pone.0046450.s002.tiff]

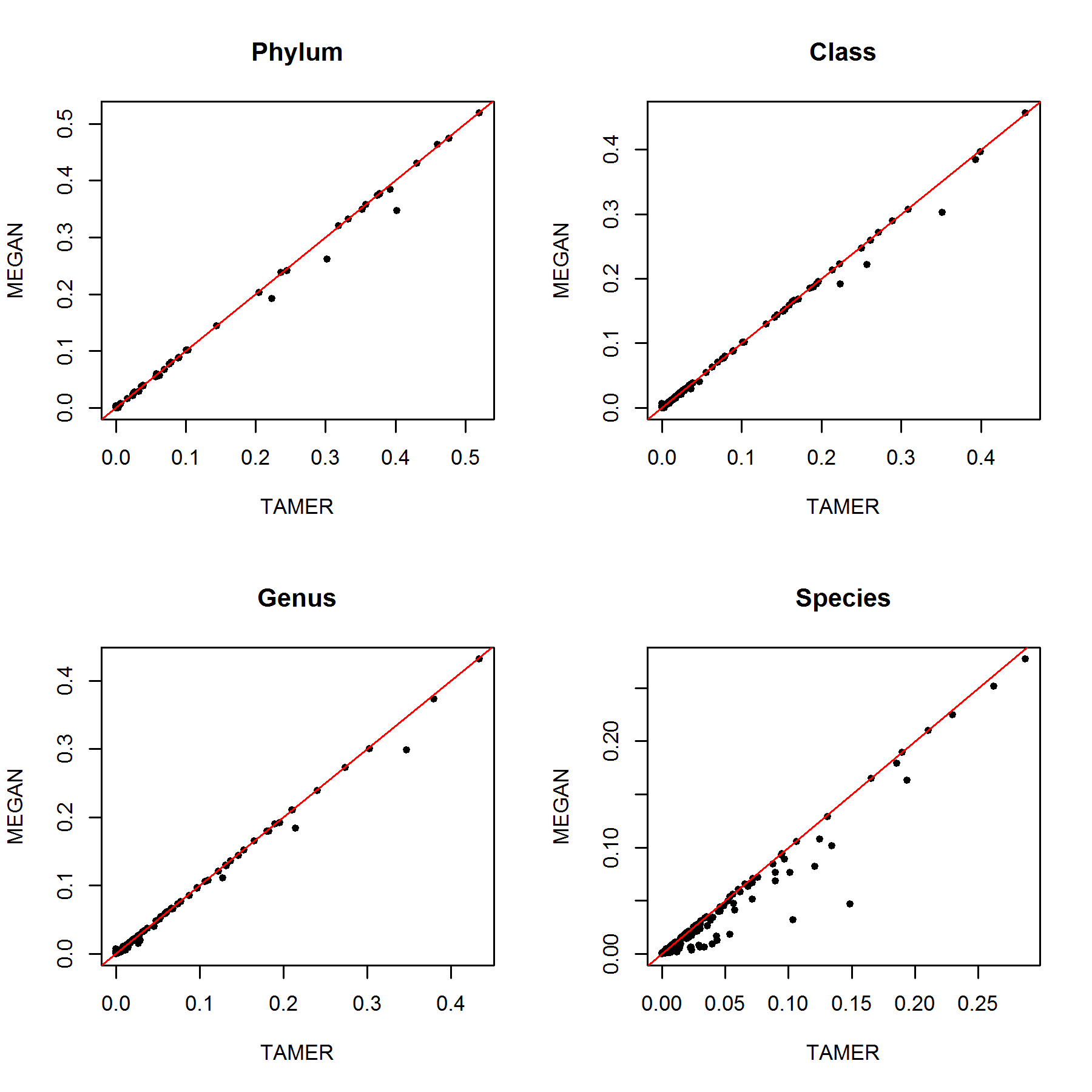

Supplement: Figure S3 — Scatter plot of estimated proportions byTAMER and MEGAN at different taxonomic ranks for the oral data. Scatter plots of estimated abundance (proportion of reads) at different taxonomic ranks by MEGAN and TAMER for all eight samples. (TIF) [file pone.0046450.s003.tiff]

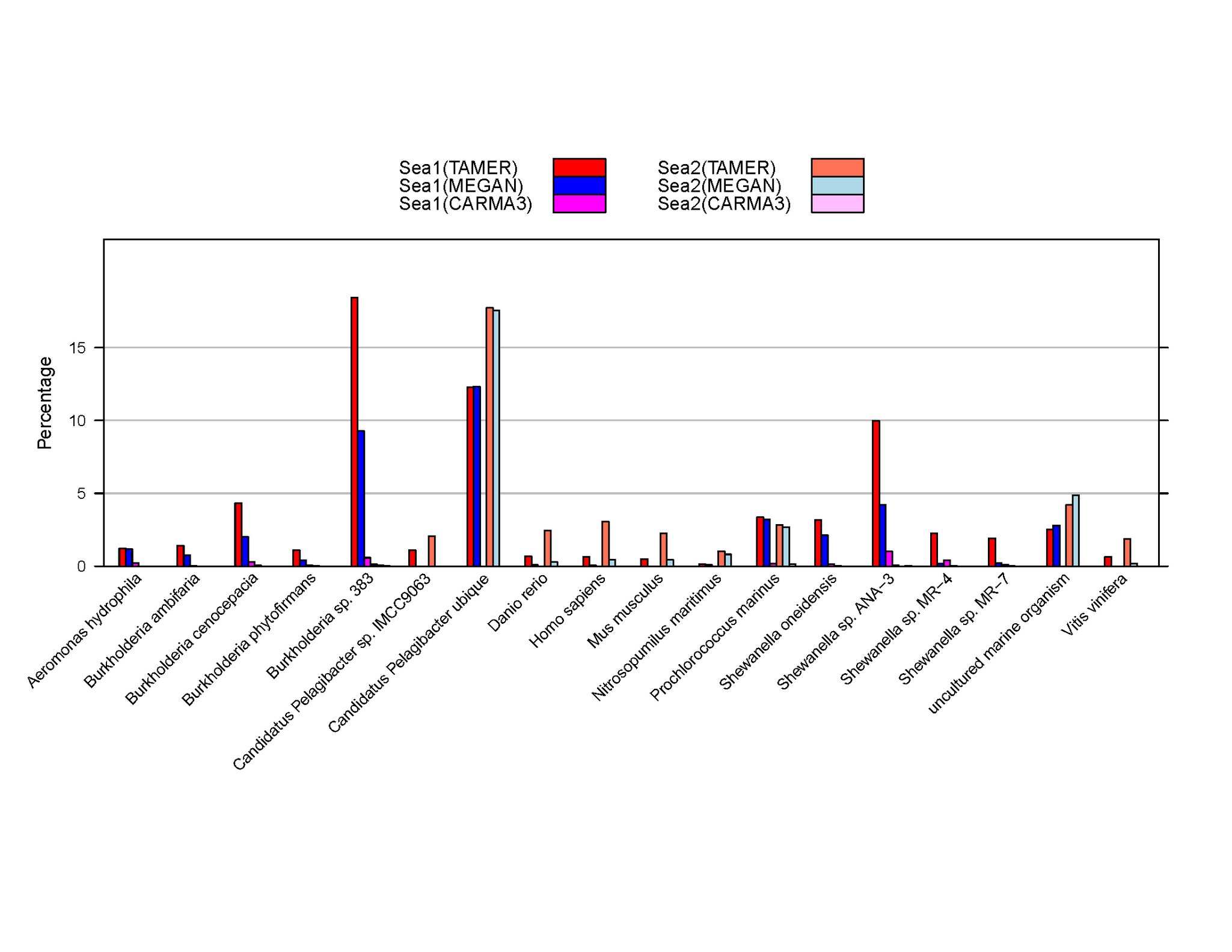

Supplement: Figure S4 — Population distribution of sea water samples at rank Species. Proportions of reads assigned to the taxa at rank Species using TAMER, MEGAN and CARMA3 are compared for the sea water datasets. (TIFF) [file pone.0046450.s004.tiff]

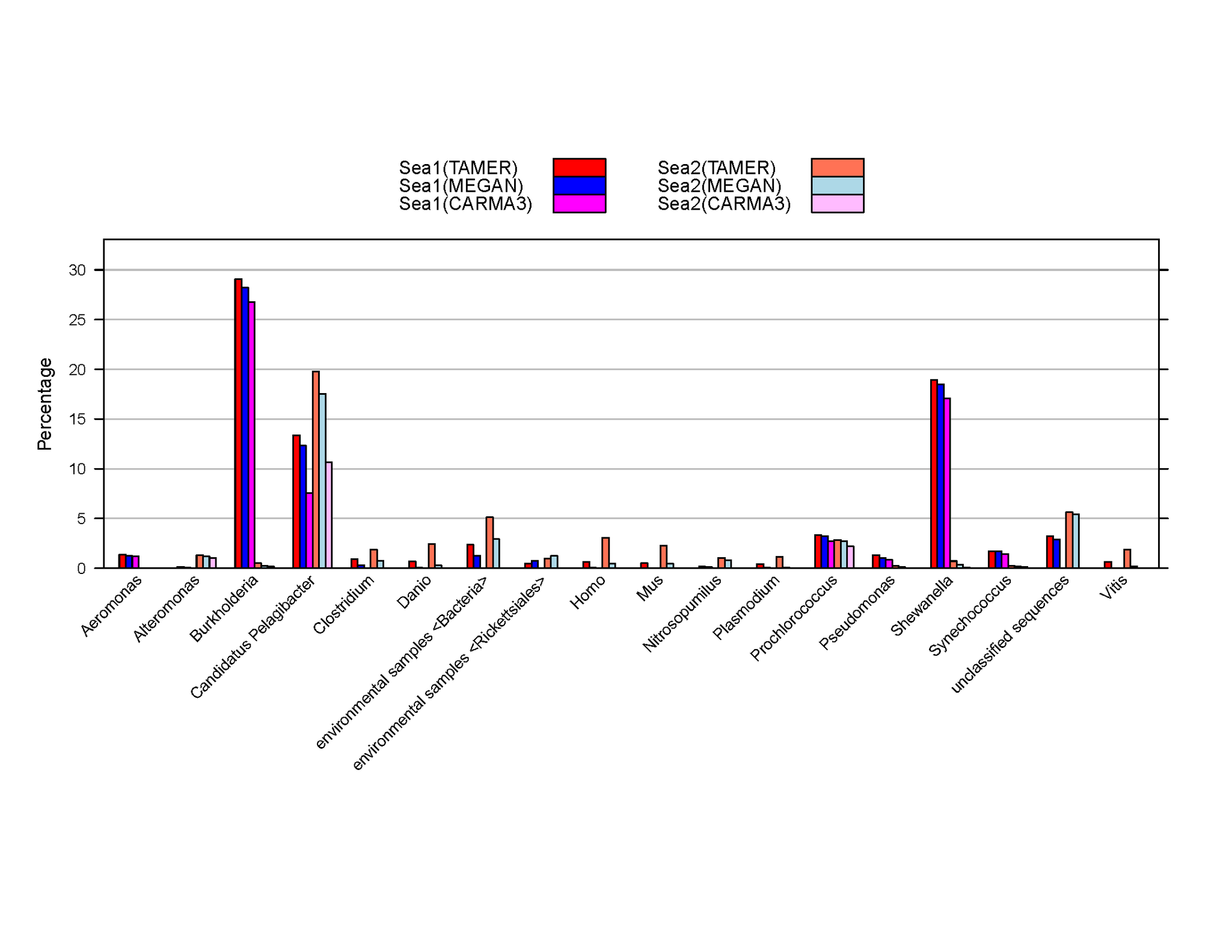

Supplement: Figure S5 — Population distribution of sea water samples at rank Genus. Proportions of reads assigned to the taxa at rank Genus using TAMER, MEGAN and CARMA3 are compared for the sea water datasets. (TIFF) [file pone.0046450.s005.tiff]
