# Supplementary material for: A Statistical Framework for Accurate Taxonomic Assignment of Metagenomic Sequencing Reads
Source: PLoS One. 2012 Oct 1;7(10):e46450. doi: 10.1371/journal.pone.0046450 (PMC3462201; doi:10.1371/journal.pone.0046450)
Supplement: Table S2 — Results for simulation study 1 with average read length of 400 bp. The percentage of correctly (TP) and incorrectly (FP) assigned reads out of total 10,000 reads with average read length of 400 bp at different taxonomic ranks using TAMER and MEGAN for simMC and simHC datasets. (DOC) [file pone.0046450.s007.doc]

|  | simMC | | | | |  | simHC | | | | |
| --- | --- | --- | --- | --- | --- | --- | --- | --- | --- | --- | --- |
|  | TAMER | |  | MEGAN | |  | TAMER | |  | MEGAN | |
|  | TP | FP |  | TP | FP |  | TP | FP |  | TP | FP |
| Species | 99.90 | 0.10 |  | 71.36 | 0.04 |  | 99.98 | 0.01 |  | 98.70 | 0.00 |
| Genus | 99.91 | 0.08 |  | 76.53 | 0.05 |  | 99.99 | 0.00 |  | 99.46 | 0.00 |
| Family | 99.99 | 0.00 |  | 98.91 | 0.00 |  | 99.99 | 0.00 |  | 99.67 | 0.00 |
| Order | 99.99 | 0.00 |  | 98.95 | 0.00 |  | 99.99 | 0.00 |  | 99.80 | 0.00 |
| Class | 99.99 | 0.00 |  | 99.07 | 0.00 |  | 99.19 | 0.00 |  | 99.05 | 0.00 |
| Phylum | 99.99 | 0.00 |  | 99.13 | 0.00 |  | 99.99 | 0.00 |  | 99.92 | 0.00 |
| Kingdom | 100.00 | 0.00 |  | 99.25 | 0.00 |  | 99.99 | 0.00 |  | 99.92 | 0.00 |
